# Real-Time PCR for Diagnosing and Quantifying Co-infection by Two Globally Distributed Fungal Pathogens of Wheat

**DOI:** 10.3389/fpls.2018.01086

**Published:** 2018-08-09

**Authors:** Araz S. Abdullah, Chala Turo, Caroline S. Moffat, Francisco J. Lopez-Ruiz, Mark R. Gibberd, John Hamblin, Ayalsew Zerihun

**Affiliations:** ^1^Centre for Crop and Disease Management, School of Molecular and Life Sciences, Curtin University, Bentley, WA, Australia; ^2^Institute of Agriculture, University of Western Australia, Crawley, WA, Australia

**Keywords:** co-infections, qPCR, hydrolysis probes, molecular disease diagnosis, pathogen interactions

## Abstract

Co-infections – invasions of a host-plant by multiple pathogen species or strains – are common, and are thought to have consequences for pathogen ecology and evolution. Despite their apparent significance, co-infections have received limited attention; in part due to lack of suitable quantitative tools for monitoring of co-infecting pathogens. Here, we report on a duplex real-time PCR assay that simultaneously distinguishes and quantifies co-infections by two globally important fungal pathogens of wheat: *Pyrenophora tritici-repentis* and *Parastagonospora nodorum*. These fungi share common characteristics and host species, creating a challenge for conventional disease diagnosis and subsequent management strategies. The assay uses uniquely assigned fluorogenic probes to quantify fungal biomass as nucleic acid equivalents. The probes provide highly specific target quantification with accurate discrimination against non-target closely related fungal species and host genes. Quantification of the fungal targets is linear over a wide range (5000–0.5 pg DNA μl^-1^) with high reproducibility (RSD ≤ 10%). In the presence of host DNA in the assay matrix, fungal biomass can be quantified up to a fungal to wheat DNA ratio of 1 to 200. The utility of the method was demonstrated using field samples of a cultivar sensitive to both pathogens. While visual and culture diagnosis suggested the presence of only one of the pathogen species, the assay revealed not only presence of both co-infecting pathogens (hence enabling asymptomatic detection) but also allowed quantification of relative abundances of the pathogens as a function of disease severity. Thus, the assay provides for accurate diagnosis; it is suitable for high-throughput screening of co-infections in epidemiological studies, and for exploring pathogen–pathogen interactions and dynamics, none of which would be possible with conventional approaches.

## Introduction

Co-infections, whereby a host-plant is invaded by multiple pathogens or multiple strains of the same pathogen, are common in the field and can have major consequences for disease ecology and pathogen evolution (Alizon et al., 2013). Despite the recognition of the significance of co-infection (Savary and Zadoks, 1992; Zadoks, 1999; Tack et al., 2012), empirical studies are still few, due mainly to the complexity of distinguishing/quantifying multiple pathogens, which often requires suitable molecular tools (Tollenaere et al., 2012; Tollenaere et al., 2016). As a result, the theoretical understanding of co-infection has largely outpaced experimental studies in natural and agricultural systems (Hood, 2003; Abdullah et al., 2017) and our capacity to understand and manage the processes and consequences of co-infection is limited until current and future theoretical models can be validated by reliable data.

Methods used for distinguishing multiple pathogens from co-infected tissues involve detection of nucleic acid targets using metagenome sequencing and/or analysis of melt-curves from PCR primers with various annealing properties (Brandfass and Karlovsky, 2006; Tollenaere et al., 2012; Gelaye et al., 2017). Such methods enable multiple pathogen detections but provide limited quantitative information on the relative abundance of each pathogen and their effects on overall disease (Bernreiter, 2017). The quantification of pathogen abundance is critical, as many pathogens are naturally present within plants, but their infection levels, pathogenicity and hence relative impacts can differ vastly (Bakker et al., 2014). At present, real-time quantitative PCR (qPCR) is the most reliable technique for measuring disease load in a sample while providing species specificity (Holland et al., 1991; Bates et al., 2001; Schena et al., 2006). Successful qPCR-based multiplexed assays have been developed for detection of *Phytophthora* diseases of soybean (Rojas et al., 2017), but the accuracy of quantification is often compromised in samples with unbalanced target ratios (Klerks et al., 2004; Atallah et al., 2007). Thus, to ensure reliable quantification, co-infection studies often analyze the abundance of multiple pathogens in separate reactions. Due to the cost of labor and resources required, this approach is limited to small-scale investigations limiting our capacity to investigate co-infections over large-scale host populations.

In order to address the limitations of current approaches, we investigate the use of dual-labeled species-specific probes to simultaneously detect and quantify two globally distributed fungal pathogens of wheat: *Pyrenophora tritici-repentis* and *Parastagonospora nodorum*. These two foliar fungal pathogens cause tan (yellow) spot and septoria nodorum blotch in wheat, respectively, damaging photosynthetically active leaf area and causing substantial yield losses (Bhathal et al., 2003). Symptoms caused by these two diseases are difficult to distinguish and may be misdiagnosed as other unrelated physiological abnormalities (Moffat et al., 2015), creating a challenge for conventional disease diagnosis and subsequent management strategies. Symptoms can also vary markedly depending on host genotype, further hampering accurate disease diagnosis (Lamari and Bernier, 1989). Interestingly, *P. tritici-repentis* carries a pathogenicity/virulence gene, *ToxA*, thought to have been acquired laterally from *Pa. nodorum* (Friesen et al., 2006). This suggests that co-infection by these two fungi is likely to occur in nature and may have consequences for disease management strategies. Despite the high likelihood of co-infection by these two fungi and difficulties of identification, no tools have been developed for the diagnosis of their abundance in co-infected host materials.

Here, we report a duplex assay that distinguishes specific DNA sequences unique to each of these fungi and quantifies their presence by direct comparison to standards amplified in parallel reactions. The basis of this method is the uniquely assigned fluorogenic reporters for each species sequence. The fluorogenic reporters, when bound to the target, quantify fungal b as nucleic acid equivalents. We report on a series of experiments designed to demonstrate the specificity and sensitivity of the method using both a simulated genomic DNA matrix of both fungi at varying ratios as well as naturally infected wheat leaves collected from the field. We also examine the capacity of the technique to show that a species that is present at very low level can be detected and quantified, with very limited interference, in the presence of an abundant species. Finally, we correlate the amount of fungal DNA quantified using the duplex qPCR assay developed here with the level of infection measured by conventional disease scores.

## Materials and Methods

### PCR Primers and Conditions

*P. tritici-repentis* primers were designed to target a species-specific multicopy genomic region described previously (Moffat et al., 2015; See et al., 2016). Briefly, a 4.65-kb region of the *P. tritici-repentis* isolate Pt-1CBFP carrying the *ToxA* gene (supercontig_1.4) was aligned to an orthologous genomic region of the *Pa. nodorum* isolate SN15 (scaffold_55). All known isolates of the targeted fungi were included in this step to ensure that the primers amplify all known isolates of the pathogens of interest. A pair of primers that target a 99-bp fragment located 701-bp upstream of the *ToxA* coding region was designed to detect *P. tritici-repentis* (**Table 1**). These primers amplify a short fragment (99-bp) within the promoter region of a low molecular weight host-selective toxin. This multicopy region has been detected in a number races of *P. tritici-repentis* isolates collected from around the world (Moolhuijzen et al., 2018). The size of the primer pair was restricted to 99-bp to ensure comparable amplicon sizes between *P. tritici-repentis* and *Pa. nodorum*.

**Table 1:** List of the oligonucleotides used in this study.

Sequence ID^∗^	5′→3′ sequence	Product length (bp)	GC (%)
*Ptr*–Forward	GTCTCCTCTGGTGGTATG	99	55.6
*Ptr*–Reverse	GCTCTTAGTGAAGTTCAATC		
*Ptr*–Probe	TACCTCTACTCGGTCGCCTATGG		
*Pn*–Forward	**ACCGCATTACCAAAGTTC**	112	45.8
*Pn*–Reverse	**ACTGGAACTGGAACAATAAG**		
*Pn*–Probe	CCTGAATGCTCTTGACACTTGGTT		

^∗^*Ptr and *Pn* refer to *P. tritici-repentis* and *Pa. nodorum*, respectively. Sequences in bold are pre-published primers modified from Oliver et al. (2008). Other sequences are designed in this study. Primers and probes were designed to amplify short fragments of the targeted pathogen DNA*.

*Pa. nodorum* DNA was amplified using primers modified from those previously described by Oliver et al. (2008). Additional 3-bp was included at the beginning of each primer sequence to allow the probe to overcome the issue of primer–dimer association (Barbisin et al., 2009). *Pa. nodorum* primers amplify a 112-bp fragment of a highly conserved anonymous gene (SNOG_01116⋅1). This gene has no significant similarity to any other sequences in the publically available genome databases (Oliver et al., 2008). All primers were designed using OligoArchitect^TM^ primer analyzer (Sigma, Life Science) and scanned against the National Center for Biotechnology Information GenBank database using basic local alignment search tools to ensure their specificity.

The designed primers were subjected to conventional PCR to confirm their specificity. Each PCR reaction contained 1xMyTaq buffer, 250 nM forward primer, 250 nM reverse primer, 1 unit MyTaq DNA polymerase (Bioline), and 5 ng DNA template. Reactions were performed as follows: 3 min initial denaturation at 95°C, 35 cycles of 30 s denaturation at 95°C, 30 s of annealing at 58°C and 1 min extension at 72°C. Electrophoresis of PCR products was performed on 2% agarose gels stained with SybrSafe (Life Technologies) and visualized under UV light. Reproducibility of the results was confirmed by running the PCR with negative controls in duplicates. The PCR step also included DNA samples from five common fungal pathogens of cereals as negative controls. Colonies of *Pyrenophora teres* f. sp. *maculata*, *Pyrenophora teres* f. sp. *teres*, *Blumeria graminis* f. sp. *tritici*, *Alternaria alternata*, and *Fusarium graminearum* were kindly provided by Steven Chang, Curtin University/Centre for Crop and Disease Management. Species identity was confirmed by sequencing the internal transcribed spacer of the ribosomal DNA (Schoch et al., 2012). All sequence analyses and multiple sequence alignments were carried out using Geneious version R6.1.6.

### Real-Time PCR Probes and Conditions

Two dual-labeled probes were custom-designed and assigned to hybridize with a complementary region between the forward and the reverse primers. The *P. tritici-repentis* and *Pa. nodorum* probes were 23-bp and 20-bp in length, respectively. The length of each probe was chosen to ensure probe-primer hybrids are formed in a complementary manner with the length of the primers. Each probe was labeled with a unique fluorogenic reporter to ensure that target sequences of both pathogens were amplified simultaneously but detected independently. The *P. tritici-repentis* probe was labeled with 6-carboxyfluorescein (FAM^TM^; Sigma-Aldrich). The *Pa. nodorum* probe was labeled with CAL Fluor Gold^®^ (CFG; Sigma-Aldrich). FAM has emission maxima between 494 nm and 518 nm and CFG emission peaks between 538 nm and 559 nm. The fluorogenic reporters were selected based on the capacity of the CFX96 detection system, the instrument used in this study, to resolve overlapping spectra. This was determined prior to carrying out the experiments using a spectra overlay tool available at http://www.Qpcrdesign.com/spectral-overlay. Both probes were paired with the non-fluorescent black hole quencher-1 (BHQ-1^®^; Sigma-Aldrich).

Probes and their matching primers were run on a 96-well spectrofluorometric thermal cycler (Bio-Rad CFX96) with the following conditions: 15 min at 95°C, 15 s denaturation at 95°C, 20 s at 72°C followed by 40 cycles of 15 s at 95°C and 30 s at 58°C. Each 20 μl reaction volume contained 5 μl of the sample and 12 μl iQ^TM^ Multiplex Powermix (Bio-Rad). For a fixed amount of target template, *P. tritici-repentis* DNA was amplified faster than *Pa. nodorum* DNA. Hence, probe and primers of *P. tritici-repentis* were restricted to obtain comparable quantification cycles (Cq) to that of *Pa. nodorum*. In a preliminary experiment, the concentrations of primers and probes were gradually increased from 50 up to 450 nM at 50 nM intervals. *P. tritici-repentis* DNA was amplified using 200 nM forward primer, 200 nM reverse primer and 100 nM probe. *Pa. nodorum* DNA was amplified using 250 nM forward primer, 250 nM reverse primer and 150 nM probe. Unless specified, reactions were carried out in duplex where primers and probes for both species were applied together. A preliminary experiment showed a comparable amplification efficiency between duplex and singleplex reactions and no evidence of cross-amplification among primers and probes of the two species was observed (**Supplementary Table 1**). Presence of any non-specific amplicon was examined using post-PCR melt curve analysis.

### DNA Extraction and Quantification

Pure genomic DNA from fungal colonies was extracted using the Bio-sprint 15 plant DNA kit (Qiagen) as per the manufacturer’s protocol. Fungal cultures were maintained on agar plates as described elsewhere (Moffat et al., 2015). Mycelia were harvested from these cultures and ground into a fine powder in liquid nitrogen. Subsamples (40 mg ground tissue) were placed into 1.5-ml microtubes, which were then used for DNA extraction. The concentration of DNA in each subsample was determined using a NanoDrop 2000 UV-Vis spectrophotometer (Thermo Scientific) and diluted to 50 ng μl^-1^ in ultrapure PCR grade water. DNA was stored at -20°C until used. Aerosol protected pipette tips were used throughout the extraction and quantification steps to prevent DNA contamination. DNA from infected wheat leaves was extracted using the same DNA extraction kit and quantified as described above.

### DNA Spiking and Field Validation

In two independent experiments, a fixed concentration of fungal DNA of one pathogen (5 ng μl^-1^) was spiked into a progressively decreasing DNA concentration of the other pathogen (5, 0.5, 0.05, and 0.005 ng DNA μl^-1^). This generated a ratio of DNA concentration from one pathogen to the other in the sample ranging from 1:1 down to 1:10000. The spiking aimed to simulate the analysis of samples derived from infection conditions whereby the two species occur at different relative abundance, which is typical of many foliar fungal infections. Recovery of fungal DNA for the respective pathogens was expressed as the ratio of the total concentration of fungal DNA quantified using the qPCR method to the amount of DNA added × 100.

In a third experiment, recovery of fungal DNA was measured in fungal DNA samples spiked with abundant wheat DNA. The concentration of fungal DNA in the samples was progressively decreased from 5 down to 0.05 ng μl^-1^ while background wheat DNA was increased from 5 to 100 ng μl^-1^. The experiment simulated analytical conditions where fungal DNA is present in small concentrations against a background of ample wheat DNA, such as would occur where infection severity and/or incidence are low.

To evaluate how well the assay works for field samples, diseased leaves from the wheat variety Scout were collected from a site in the southwest of Western Australia (31°.74S, 116°.70E). Scout is rated as susceptible to very susceptible to both *P. tritici-repentis* and *Pa. nodorum* (DAFWA, 2016). Sampled leaves were visually inspected for diseased leaf area and given scores on 0-100 scale. Leaves were then split into two groups; one group (*n* = 9) was surface-sterilized in 2% chlorine and incubated on agar Petri-dishes in an attempt to characterize the causal agent of the disease. Leaves from the second group (*n* = 9) were ground in liquid nitrogen and used for DNA extraction. 50 ng μl^-1^ gDNA from the infected leaves, along with 50 ng μl^-1^ gDNA from uninfected leaves from glasshouse-grown plants (negative controls), were first analyzed by conventional PCR. A further 50 ng μl^-1^ gDNA from the same infected leaves and controls were then analyzed using qPCR.

### Detection and Quantification of Fungal Biomass

Fluorescence data from the qPCR machine were retrieved during the annealing step of every Cq. Threshold fluorescence was set automatically by the instrument manager system (CFX Manager Version 2.0.8) before carrying out the assay. A log-linear standard curve of a 10-fold dilution series corresponding to 5000 to 0.5 pg μl^-1^ was generated by plotting the logarithms of known concentrations of fungal DNA against the Cq values. The resulting regression equations were used to calculate fungal DNA in unknown samples. No-template controls, where sterile water was added instead of DNA, were included in each reaction. Limit of detection of the duplex assay, the lowest concentration at which reliable detection can be achieved, was determined following Armbruster and Pry (2008). All reactions were run with three replicates and samples that gave positive fluorescence before no-template controls were considered positive.

## Results

### Specificity of the Assay

**FIGURE 1:**
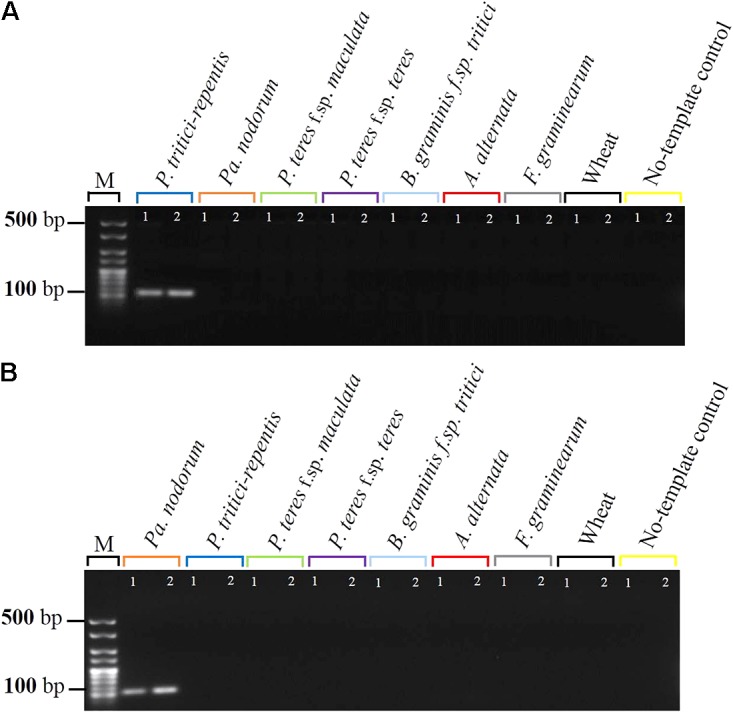
Specificity testing of primers by agarose gel electrophoresis. **(A)** Primers of *P. tritici-repentis, Ptr*-Forward and *Ptr*-Reverse, were tested against *Pa. nodorum* DNA, and **(B)** primers of *Pa. nodorum*, *Pn*-Forward and *Pn*-Reverse, were tested against *P. tritici-repentis* DNA in two separate PCR reactions. Reactions included negative controls of DNA samples from five cereal fungal pathogens. Reactions including positive and negative controls were electrophoresed on 2% agarose with two technical replicates.

To test how well *P. tritici-repentis* and *Pa. nodorum* could be distinguished from each other, as well as from other common wheat pathogens and the host, we carried out two conventional PCR assays each using 5 ng gDNA. Five non-target controls of DNA from closely related cereal fungal pathogens and DNA from the wheat cultivar Scout were included in this step. Reactions were run in separate wells (i.e., singleplex). Conventional PCR provided amplicons of the expected sizes, and *P. tritici-repentis* and *Pa. nodorum* were distinguished based on the size of the amplicons (**Supplementary Figure 1**). *P. tritici-repentis* and *Pa. nodorum* primers only amplified the respective pathogen DNA. None of the five non-target controls or DNA from wheat gave specific amplicons that could be detected by conventional PCR (**Figures 1A,B**).

**FIGURE 2:**
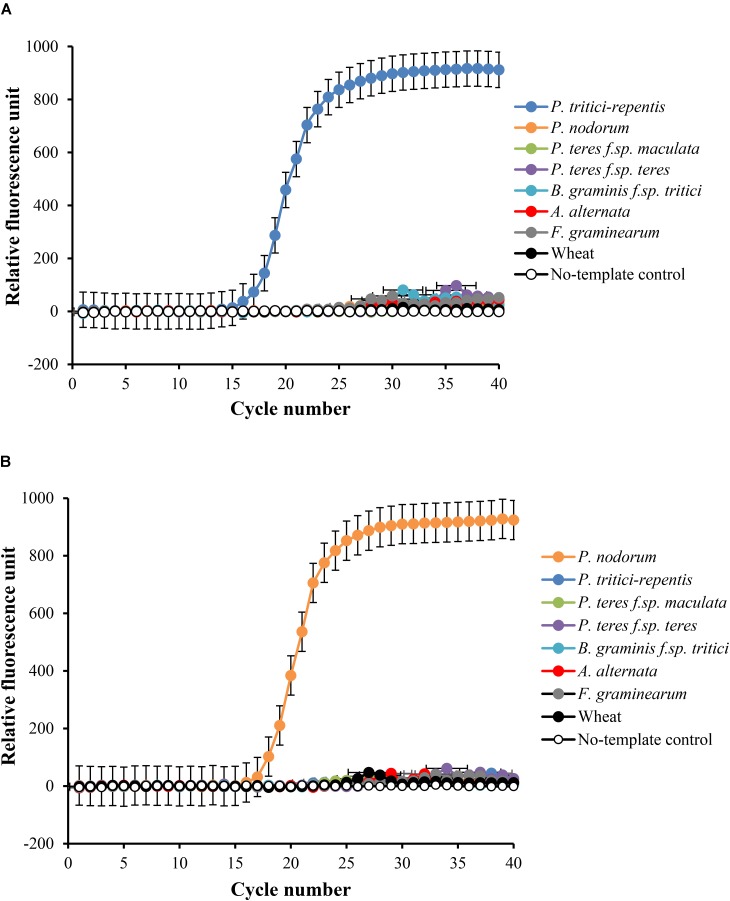
Specificity testing of primers and their matching probes in singleplex real-time qPCR settings. Amplification curve for *P. tritici-repentis* is plotted in blue **(A)** and amplification curve of *Pa. nodorum* is plotted in orange **(B)**. Primers and probes (**Table 1**) were tested against DNA from the five fungal species, a negative wheat control, and a no template control and reactions were run separately. Data represent means ± standard deviation (*n* = 3).

We then tested the specificity of the probes and their matching primers in two-singleplex real-time qPCR assays each containing 5 ng gDNA. Detection only occurred for probes that were complementary to the expected sequences, and none of the five non-target controls or the wheat DNA had specific amplification during 40 real-time qPCR cycles (**Figures 2A,B**). Samples that included no-template DNA or those that contained wheat DNA were negative during the course of the reaction (**Figures 2A,B**). *P. tritici-repentis* and *Pa. nodorum* were distinguished based on the emission spectra of the fluorogenic reporters that were used to label each pathogen probe.

### Dynamic Range, Efficiency, and Reproducibility of the Assay

**FIGURE 3:**
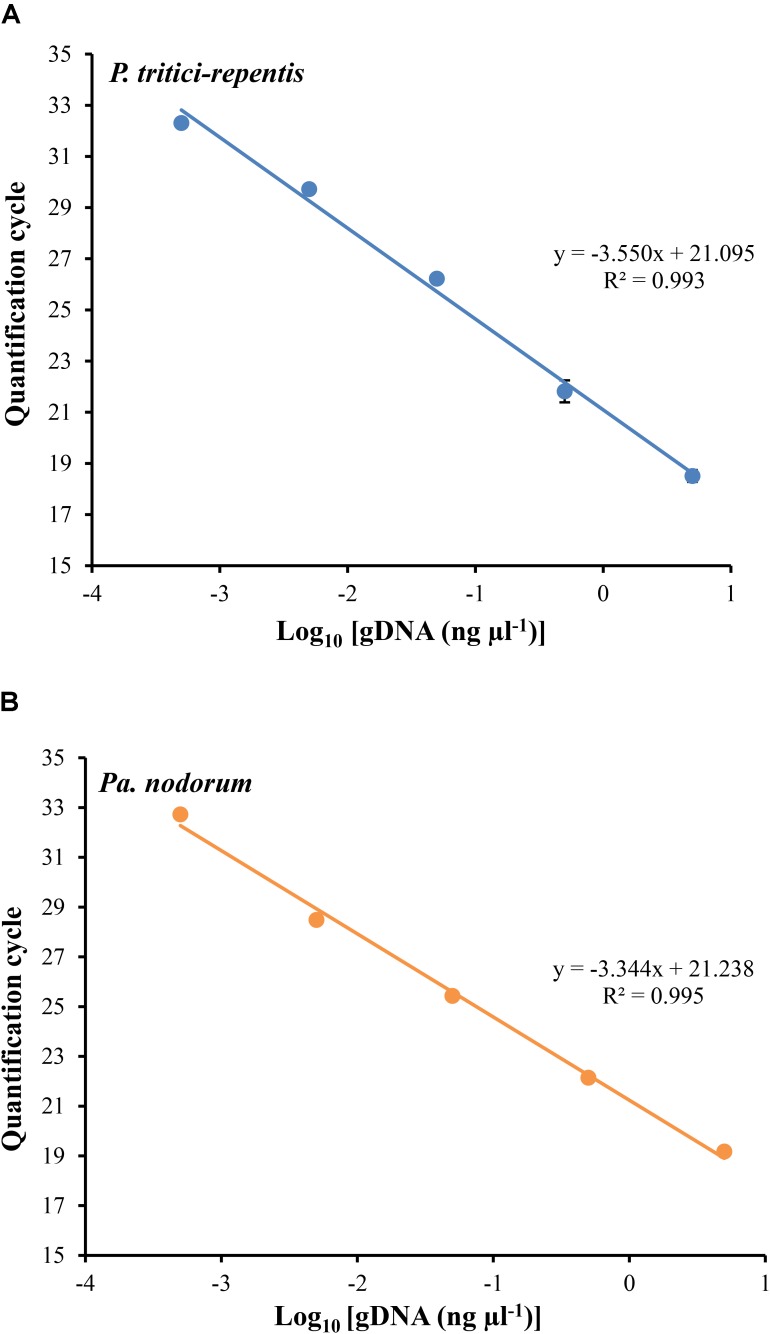
**(A)** The relationship between quantification cycle and logarithm of the concentration of fungal DNA in duplexed qPCR settings. Triplicate dilution series corresponding to gDNA concentrations of 5000, 500, 50, 5, and 0.5 pg μl^-1^ were prepared. No-template samples were included in every reaction as negative controls (*n* = 10). The quantification cycle at which fluorescent signals were observed is plotted against the logarithm of DNA concentrations of *P. tritici-repentis* **(A)** and *Pa. nodorum* **(B)**. The corresponding regression equations and coefficient of determinations (*R*^2^) are shown on the plot. Data are means ± standard deviation where visible (*n* = 3).

We carried out two duplexed standard curve experiments on DNA of *P. tritici-repentis* and *Pa. nodorum* mixed at equal ratios. A 10-fold dilution series in the range of 5000 to 0.5 pg μl^-1^ was generated, analyzed and plotted against the number of Cq required to detect fluorescence signals. Each dilution was prepared with three replicates except the no-template control, which was analyzed using 10 replicates. Fitting log-linear standard curves between Cq and fungal DNA resulted in correlation coefficients (*r*) -0.997 and -0.996 for *P. tritici-repentis* and *Pa. nodorum*, respectively. The standard curves for both species were linear over 10,000-fold dilution, and the calculated amplification efficiency was 91.28% for *P. tritici-repentis* and 99.25% for *Pa. nodorum* (**Figures 3A,B**). The slopes and intercepts of the log-linear curves were comparable between *P. tritici-repentis* and *Pa. nodorum* (*p* > 0.05). Mean Cq value (±standard deviation bars, where visible) of three replicated reactions was highly reproducible with relative standard deviation ≤ 10% (**Figures 3A,B**). The variation around the mean was independent of template concentration, and the Cq value for 10 no-template controls averaged 36.618 with a relative standard deviation of 0.35%.

### Sensitivity and Limit of Detection of the Assay

**FIGURE 4:**
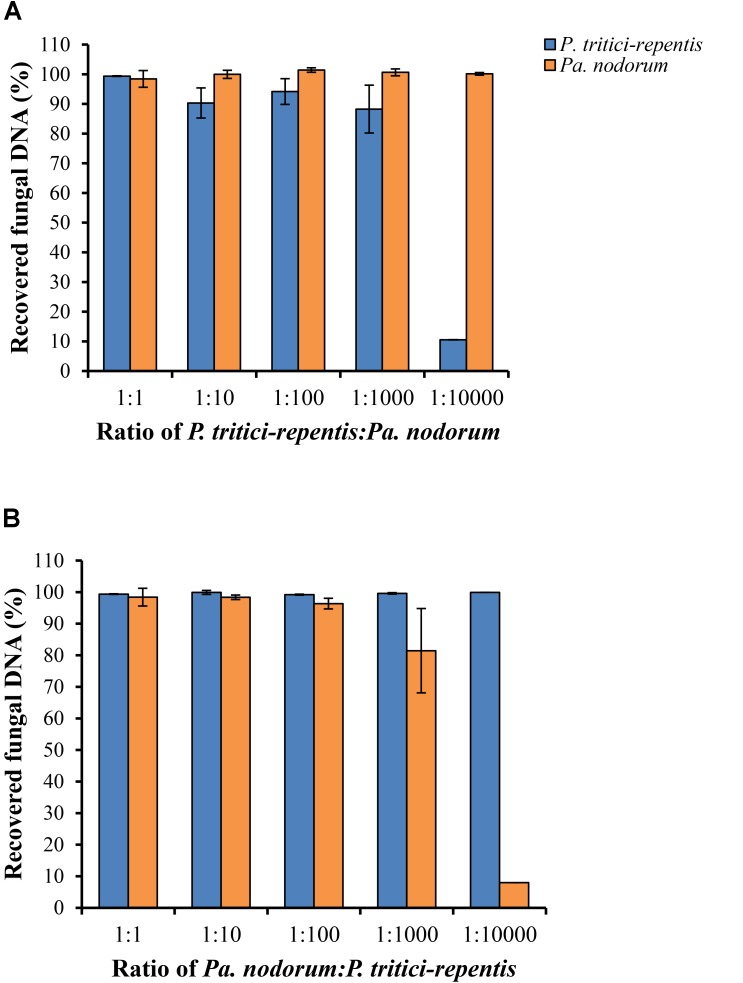
Detection and quantification of P. tritici-repentis and *Pa. nodorum* in a simulated DNA matrix of various ratios. **(A)** *P. tritici-repentis* DNA was spiked with Pa. nodorum DNA at ratios of 1:1, 1:10, 1:100, 1:1000, and 1:10000. **(B)** *Pa. nodorum* was spiked with *P. tritici-repentis* DNA at same ratios. The starting concentration was 5 ng μl^-1^ of each pathogen DNA. The subsequent ratios were sequential 10-fold dilutions (0.5, 0.05, 0.005, and 0.0005 ng μl^-1^) of one pathogen DNA, with the other held constant at 5 ng μl^-1^. Data represent means ± standard deviation (*n* = 6).

We carried out two spiking experiments using a simulated matrix of *P. tritici-repentis* and *Pa. nodorum* DNA mixed at various ratios. Results of the spiking experiments demonstrated that reducing DNA ratio of one species while keeping DNA of the other species constant did not affect the detection limit of the assay. We were able to accurately quantify fungal DNA of either pathogen down to a relative ratio of 1:100 (**Figures 4A,B**). However, further reduction of the DNA ratio to 1:1000 underestimated the amount of *P. tritici-repentis* by 11.75% and *Pa. nodorum* by 18.55%. This underestimation became more pronounced with further dilution, and at 100 ppm (i.e., DNA ratios of 1:10000), neither pathogen was quantifiable in the presence of the other (**Figures 4A,B**). The minimal DNA concentration for detection was 0.059 pg μl^-1^ for *P. tritici-repentis* and 0.036 pg μl^-1^ for *Pa. nodorum*.

**FIGURE 5:**
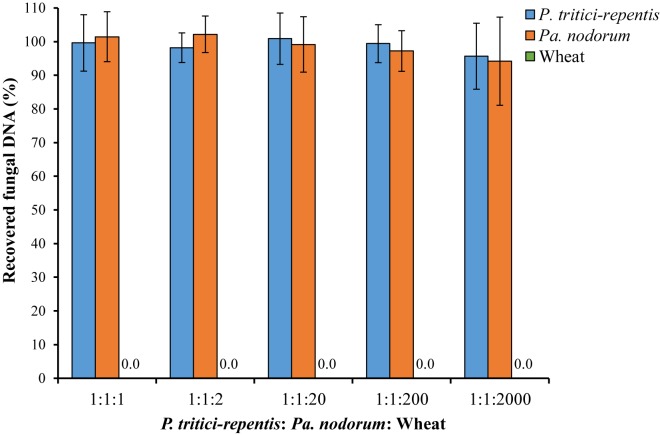
Simulated DNA matrix of various ratios of fungi to wheat DNA. Increasing concentrations (5–100 ng μl^-1^) of wheat DNA were spiked with decreasing concentrations of fungal DNA (5–0.05 ng μl^-1^). Quantification was done in real-time quantitative PCR and reactions were duplexed each containing four fungal-specific primers and two complementary probes (**Table 1**). DNA concentrations respectively were 5:5:5, 5:5:10, 5:5:100, 0.5:0.5:100, and 0.05:0.05:100 ng μl^-1^. Data represent means ± standard deviation (*n* = 6). Wheat DNA was not quantified/detected.

We also tested the ability of the assay to detect and quantify fungal DNA in a sample that included abundant wheat DNA. We were able to quantify both species with high accuracy up to a *P. tritici-repentis*: *Pa. nodorum*: wheat DNA ratio of 1:1:200 (**Figure 5**). Recovery of fungal DNA, total amount of DNA quantified using the qPCR method, was high at 95.69% for *P. tritici-repentis* and 94.20% for *Pa. nodorum* when the fungal to wheat DNA ratios dropped to 1:1:2000. At these ratios, however, relative standard deviations were 10.23% and 13.90% for *P. tritici-repentis* and *Pa. nodorum*, respectively, affecting the reliability of fungal DNA quantification.

### Field Evaluation of Naturally Infected Plants

**FIGURE 6:**
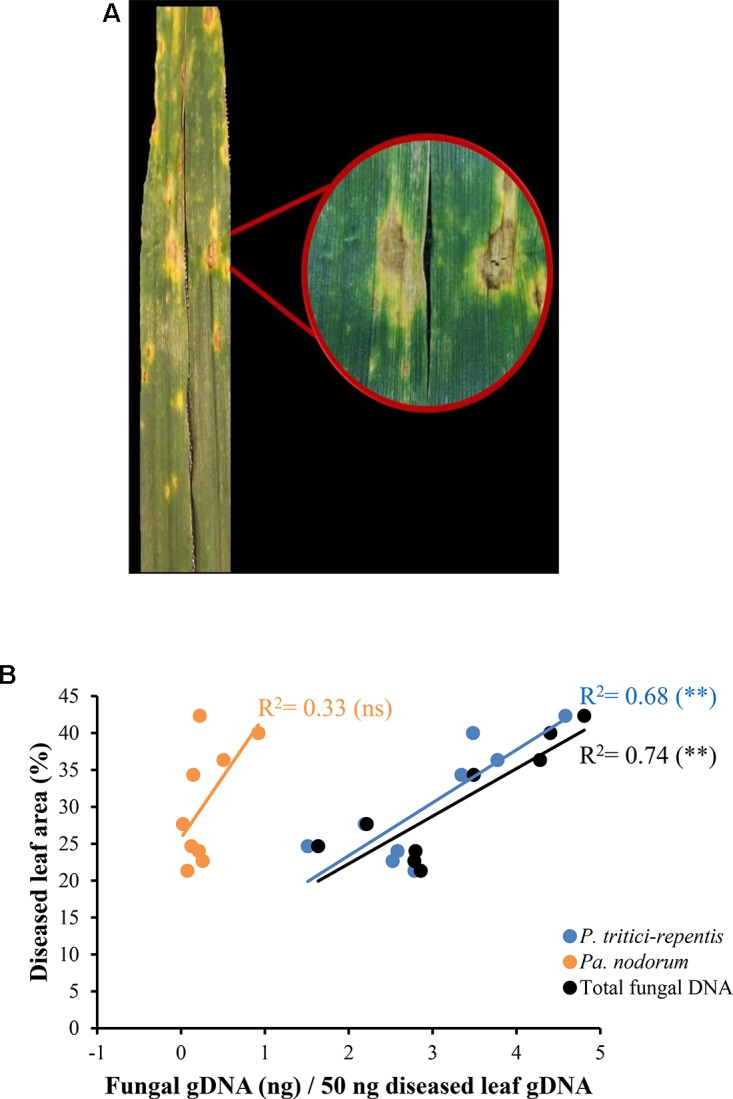
Quantification of fungal DNA in naturally infected wheat leaves. **(A)** Leaves displaying tan spot symptoms with tan necrotic centers and yellow halos. **(B)** A linear model fitted into the relationship between fungal DNA measured in real-time quantitative PCR and conventional disease score. The corresponding regression equation and coefficient of determination (*R*^2^) are shown on the plot. ns and ^∗∗^ refer to not significant and significant (*P* < 0.01), respectively.

We collected eighteen naturally infected wheat leaves from a site in the southwest of Western Australia. These leaves showed typical symptoms of tan spot with distinct yellow halos and tan chlorotic lesions (**Figure 6A**). No apparent symptoms of *Pa. nodorum* were visible on these leaves. Leaves were split into two groups; one group (*n* = 9) was incubated on agar Petri-dishes in an attempt to characterize the causal agent of the disease. Incubation on agar only yielded colonies typical of *P. tritici-repentis* (data not shown).

DNA was extracted from the second leaf group (*n* = 9) and analyzed using conventional PCR. Only one out of nine duplicated PCR reactions tested positive to *Pa. nodorum*. All reactions were positive to *P. tritici-repentis*. DNA from the same leaves that were used in the conventional PCR was then analyzed using qPCR. The duplex qPCR assay was able to detect signals corresponding to sequences of *P. tritici-repentis* and *Pa. nodorum* despite the absence of any visible symptoms of *Pa. nodorum* on these leaves (**Figure 6B**). There was a good agreement (*r* = -0.82) between total fungal DNA quantified by qPCR and conventional disease scores that were collected in the field (**Figure 6B**; black circles/line). Conventional disease scores were significantly correlated with the increase in *P. tritici-repentis* DNA, and *P. tritici-repentis* DNA was greater contributor to the disease scores than *Pa. nodorum* (**Figure 6B**; blue and orange circles/lines).

## Discussion

Quantification of the relative abundance of co-infecting pathogens requires selection of primers and probes that are compatible with each other whilst distinguishing between the co-infecting species. Recent work has identified a 235-bp multicopy region present in the *P. tritici-repentis* genome, and primers designed in this region were able to detect *P. tritici-repentis* from wheat leaves, even prior to the visible appearance of tan spot lesions (See et al., 2016). However, these primers cross-hybridized with *Pyrenophora teres* f. sp. *teres*, which the authors suggested may be overcome by the addition of fluorescence-labeled probes in the middle of the amplicon.

In this study, primers were chosen to produce relatively smaller amplicons (99–112 bp) that are more efficient for amplifying specific products than longer primers (Bustin et al., 2009). Our primers successfully distinguished targeted pathogens from non-target closely related fungal species and host genes. However, for increased specificity, fluorogenic probes were designed to hybridize to sequences within the primers enabling the assay to simultaneously distinguish and quantify DNA associated with *P. tritici-repentis* from that associated with *Pa. nodorum*. The length of the probes was designed to ensure that stable primer-probe hybrids were formed at the same annealing temperature for both species, irrespective of the length of the primers. Furthermore, probes were labeled with a unique fluorogenic-reporter allowing DNA sequences from *P. tritici-repentis* and *Pa. nodorum* to be amplified simultaneously but independent of each other. The choice of the fluorogenic reporter was decided based on instrument capability in resolving overlapping spectra. FAM-labeled probes are excited at lower emission spectra than CFG-labeled probes and hence are expected to produce stronger signals. Nevertheless, restricting the concentration of the FAM-labeled probe in the reaction optimized the fraction of the amplicon that is bound to the probe and produced comparable signals for *P. tritici-repentis* and *Pa. nodorum*. In addition to the fluorogenic reporters, probes were labeled with dark-hole quenchers to inhibit probe signals when probes are free in the solution (Holland et al., 1991). The dark-hole quencher was sufficient to inhibit signals from probes that are not in perfect contact with the target, resulting in accurate species-specific quantifications.

A series of experiments were conducted demonstrating the specificity, efficiency, and reproducibility of this assay for simultaneous detection and quantification of *P. tritici-repentis* and *Pa. nodorum*. The method reported here resolved two signals each unique to the assayed fungi. No cross-amplification with either host plant DNA or DNA from other closely related fungal pathogens of cereals was observed. The assay amplified the targeted sequences with high specificity, and we were able to detect the presence of *P. tritici-repentis* at 0.059 pg μl^-1^ and *Pa. nodorum* at 0.036 pg μl^-1^ in mixed fungal DNA matrices. Furthermore, in mixed gDNA matrices of both targeted species, the assay was able to detect fungal signals at a relative abundance as low as 1 in 10,000 with ≤10% RSD. Similarly, the assay was able to quantify the presence of *Pa. nodorum* DNA down to 23-pg in leaf samples naturally co-infected by abundant *P. tritici-repentis* up to 2192-pg. Nonetheless, there was a slight interference to the quantification of the less abundant target when DNA of the other target and/or wheat DNA was highly abundant. This may occur because reaction components including DNA polymerase, dNTPs, and MgCl_2_ become limiting in later qPCR cycles. The more abundant target may compete for reaction components with the less abundant target, delaying its amplification (Bustin et al., 2009). As a result, the quantification of the less abundant target, although remaining specific and within the detection limit, may be compromised. One way to minimize this interference is to optimize concentrations of reaction components sequentially (Bustin et al., 2009). Regardless, the assay is highly stable and reproducible. The reproducibility was confirmed by the small standard deviations of triplicates samples in the standard curves (≤10%). The amplification curves of replicate runs showed strong overlap indicating stability and reproducibility.

A limitation of most nucleic acid-based quantification assays is an inability to distinguish between DNA from living and dead cells (Pinheiro et al., 2016). Although fungal cell viability was not assessed in this work, the high correlation coefficient, 0.826, especially between *P. tritici-repentis* DNA and the conventional disease score, suggests that viability may not have a significant impact on measuring disease as DNA equivalent. Quantification of fungal DNA has been shown to provide accurate measurement of disease severity for *P. tritici-repentis* (See et al., 2016) and *Pa. nodorum* (Oliver et al., 2008). Another limitation more specific to qPCR is that only a few targets can readily be accommodated in a single homogeneous assay. The number of targets that can be fitted in one assay depends on the design of the assay and the ability of the instrument to resolve overlapping spectra. The instrument used in this study has a blue monochromatic laser source for the excitation of fluorophores, and can simultaneously detect five targets on five different channels. This limits the fluorophores that could be efficiently excited by the blue laser to those that are distant from the far-red wavelengths. Instruments with high resolution-detectors and variable-wavelength light sources may allow more targets to be included in an assay. Instruments with such specifications are available and can be employed for detection and quantification of large-scale screening of co-infection by multiple pathogens.

Detection of multiple pathogens has also been achieved by the use of post-PCR dissociation curve analysis. Such methods use a set of primers with different melting temperatures and G + C contents. Using this technique up to 10 microbial pathogens of humans have been distinguished in a single assay (Gelaye et al., 2017; Jiang et al., 2017). Post-PCR methods, although specific and accommodating several targets in a single assay, do not provide quantitative information on the level of disease. Plant tissues can be inhabited by a large number of pathogenic and non-pathogenic microbes. Nevertheless, infection levels of these microbes and the damage they cause to the plant can vary greatly (Bakker et al., 2014). Our assay offers several advantages over the dissociation curve analysis and many other methods for diagnosis of co-infection. First, no post-reaction processing is required. Reactions are driven to completion allowing higher levels of sensitivity even when target sequences are present at low concentrations. Second, the assay provides quantitative data on the level of disease load in a sample, which other methods including metagenome sequencing do not offer. Finally, the cost-effectiveness, although not directly estimated, of testing multiple agents in a single test allows more testing using the same amount of reagents and staff time. When coupled with a 96-well capacity, this assay offers a sensitive and quantitative high throughput methodology for detection of co-infection in plants. The method can also be used to study epidemiological consequences of co-infection in the field.

## Author Contributions

AA contributed to the conception of the idea, defined/selected the primers, conducted the experiments, analyzed the data, and drafted the manuscript. CT assisted in setting up the conventional PCR and testing of the primers. CM, FL-R, AZ, MG, and JH supervised the work and provided critical suggestions on the article. All authors read and approved the final version of the article.

## Conflict of Interest Statement

The authors declare that the research was conducted in the absence of any commercial or financial relationships that could be construed as a potential conflict of interest.
