# Supplementary material for: Real-Time PCR for Diagnosing and Quantifying Co-infection by Two Globally Distributed Fungal Pathogens of Wheat
Source: Front Plant Sci. 2018 Aug 9;9:1086. doi: 10.3389/fpls.2018.01086 (PMC6095046; doi:10.3389/fpls.2018.01086)
Supplement: Supplementary file 1 [file Table_1.docx]

**Table Supplemental 1.**

| Target species | Singleplex reactions | | | | | Duplex reactions | | | |  |
| --- | --- | --- | --- | --- | --- | --- | --- | --- | --- | --- |
|  | R^2^ | Slope | Intercept | E* (%) | R^2^ | | Slope | Intercept | E* (%) | *P*-value^†^ |
| *P. tritici-repentis* | 0.997 | -3.504 | 21.018 | 92.92 | 0.993 | | -3.550 | 21.095 | 91.28 | >0.05 |
| *Pa. nodorum* | 0.996 | -3.210 | 21.305 | 104.89 | 0.995 | | -3.344 | 21.238 | 99.25 | >0.05 |

**Table Supplemental 1 caption.**

Parameter estimates and reaction efficiencies of the triplicated standard curves constructed in singleplex and duplex settings.

**Table Supplemental 1 footnote.**

^*^E refers to the calculated efficiency of the reaction (E=10^(-1/slope)^ – 1).

^†^Comparison of parameter estimates between the regressions in singleplex and duplex settings.
