# Supplementary material for: Real-Time PCR for Diagnosing and Quantifying Co-infection by Two Globally Distributed Fungal Pathogens of Wheat
Source: Front Plant Sci. 2018 Aug 9;9:1086. doi: 10.3389/fpls.2018.01086 (PMC6095046; doi:10.3389/fpls.2018.01086)
Supplement: Supplementary file 2 [file Image_1.pdf]

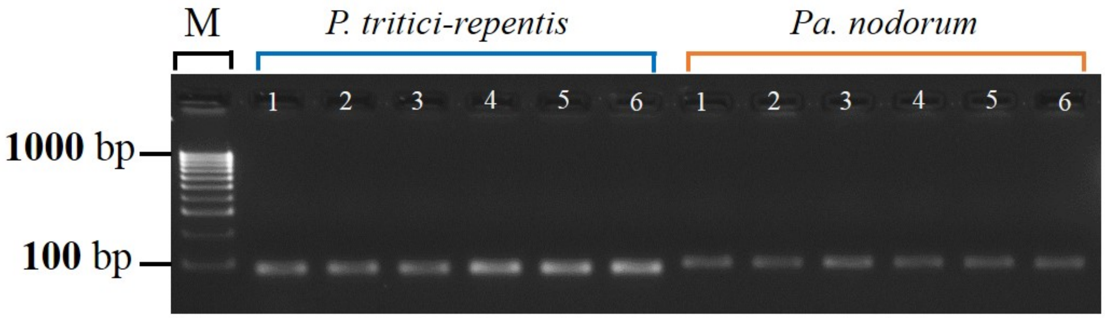

Supplementary figure 1. PCR testing of *P. tritici-repentis* amplicons (99-bp) as compared in size to *Pa. nodorum* amplicons (112-bp). Reactions were run in six replicates.
